# Roserl

**Published:** 1876-09

**Authors:** Marie Orm


*From Treasure Trove.*

### *ROSERL.*

### AN ALPINE SCENE.

---

BY MARIE ORM.

---

MOUNTAIN on mountain rising one behind the other, most of them starting out of dark forests, in wild fantastic forms; some, skirted at the bottom by woods and fields, crowned with the bare stone fir, its outline sharply drawn against the blue sky, presenting to view a gradual cessation of vegetation. Large extents of grassy ground lying between the rocky and woody chaos, hidden and unsuspected; though they did not the little white Alm huts, peeping out here and there, and occasionally the sound of the cow-bells, betray their existence. To complete the picture, a glimpse of the row of glaciers in the background.

On one of those softly-turfed, wood-bound, rock-adorned Alms stood Roserl's sennerhulte, looking down from its lofty position into Bavaria on one side, on the other into Tyrol. The small, white-washed hut with its brown door and glistening window; the wooden loft above, under the overhanging roof of shingles, the latter kept down by weighty stones, placed in regular rows upon them; with its adjoining cow-house, presented altogether a most picturesque aspect. And climbing round and round those rugged rocks, following the narrow, well-trodden path, one might approach the hut and behold the fair inmate of that airy summer residence, Roserl herself.

Hiller's Roserl is the fairest girl of the village, nay, of the whole neighborhood. The opinion of the younger men, however, is divided between Hiller's Roserl and the Mossbauern Feferl; but the elder men, whose judgment may be taken as less biassed, and even the women, declare Roserl's charms perfection, and give her the credit of being the belle on the Alm. So, I think, we may fully justify our own assertion.

Hiller's Roserl need not have gone up as "sennerin" if she had no mind to do so. She was an only child. Her father could spare a thoosand florins and be none the poorer; her mother had a huge wardrobe, well-stocked with snow-white, self-spun linen, to which every winter saw a considerable addition; a handsome bedstead with overflowing feather beds, and silk coverlets, all in flounced cases; a goodly number of different colored neckerchiefs and aprons; a dozen heavy silver spoons; a necklace of twelve rows of silver chains, united by an immense clap, and other trinkets—all ready for Roserl, whenever she chose to transfer her beautiful self from her paternal roof to that of a husband.

Roserl, although she could have afforded to send a maid with the cows, preferred, as all girls in the Alps do, to go up herself. On no account would she have missed her "sommerfrische."

Long and dreary enough were the winter months in the narrow homes below, when the girls were left to their own resources; the boys being away in the forests, often in very distant places.

When summer work is ended, and snow has covered their fields, the young peasants leave home to look out for winter occupation as woodmen in the imperial or royal forests, taking their unemployed horses or oxen with them.

In the mountains, the felling of the trees, the logging and sledging of the timber, is attended with great danger—more so than any other profession, and yet there is no other occupation so much to the young peasant's liking. There is something inspiriting, highly pleasant, in a work so manly, calling forth so much strength and dauntlessness. There is but one thing placed higher in the Alpine youth's estimation than that kind of forest work—wild hunting.

The "wildern," though scarcely more dangerous, is still more exciting. And there are few—we may without risk say none; at least, none possessieg bodily vigor—who have not tried their hand at it. Not everyone, of course, is lucky enough to become a regular wildschutz by profession, to attain to fame in that line being the terror (and the boast at the same time) of the neighborhood, and the trouble of the forester.

Most of the youths are kept down to a certain limit in the game of wildern, and home influence often induces them to give it up early, confining themselves to an occasional shot here and there.

Well, in winter, when the boys lead their isolated and laborious life in the woods, and only occasionally appear to catch a glimpse of home, the girls have a rather dull time of it; and no wonder they greet the returning spring and the fair prospect of coming summer joys with ecstacy.

Pretty Roserl had been as eager as any of the maidens to take her fly upwards, and surely her life on the Alm was all enjoyment.

Roserl, we are sorry to say, was as great a flirt as she was pretty; and what wonder?—and where the evil? No evil until *now*.

Roserl stood leaning against the doorpost of the stable-gate, singing a pretty landler, and patting the necks of her cows, calling them caressingly by their names, as they trotted past her into the stable. By a look at the sinking sun—clock she had none—she had ascertained that it was time, and stepping upon an elevated rock near her hut, had put a hand on each side of her mouth and uttered her yodler—a call consisting of a succession of notes, the peculiar call in the Alps. A minute afterwards, one would hear the harmonious sound of the tinkling cowbells as the stragglers assembled, and then, in a long line, following one another steadily and orderly, wound their way round to their night quarters.

Sometimes, in hot weather, the cows sleep out; but when thunderstorms become frequent, the Almerin thinks it safer to see them in before nightfall.

Roserl's stables were a mere shed, just a shelter. The wide gates remained open, besides every shutter and air-hole in the roof, to make it pleasanter to the cows.

Hiller's Roserl would have been a pretty girl in any dress, but surely none could have set off her figure to more advantage than her picturesque national costume.

Her well-formed feet were dressed in a pair of black shoes and white stockings, the spotless purity of which could only be accounted for by the total absence of dust on the green Alm. Sometimes, when the girl gave herself a sudden swing, you could catch a glimpse of the bow of scarlet silk ribbon which was tied as garters under her knees; but usually her skirt, though short enough, did not display more than a considerable portion of the white, well-fitting stocking. The skirt was made of a gay print, well-starched and ironed, worn over some ample petticoats of the same material. A white muslin apron covered the whole part of the dress, tied round her waist by scarlet ribbon with long flying ends. Her black velvet bodice, laced in front with a silver chain, fitted very tightly round her slender waist, but allowed full developement to her bust. It was low and sleeveless, worn over a shirt of coarse but snow-white linen, reaching nearly up to her throat, which was encircled by a quantity of loose rows of coral beads, fastened behind with a heavy gold clasp. The short puffy sleeves, bordered by a bit of embroidery, left her round, sunburnt arms entirely bare. Two immense plaits of glossy brown hair were fastened to her head by large silver pins; and a small green felt hat with a golden tassel, sitting coquettishly on one side of her head, gave to the whole costume its peculiar character.

But under the tasselled hat, and framed into these soft rich plaits, was a face of pure white style, the regularity of whose features was set off by a tender complexion of clear red and white; by coral lips and pearly teeth, always ready to greet you with a bright, sunny smile; and a pair of such eyes! Her eyes were blue, deep blue, with a soft tenderness in the glance, but apt to take all hues and expressions, changing with every movement of her soul.

This evening, when Roserl had attended to the cows and finished her day's work in the dairy, she put on a fresh muslin apron, took up her white knitting, and stepped out on to a jutting point from whence she could command a view into two different realms. The boundary between Bavaria and Austria is evidenced by a sort of road cut through the forest, over the hills, through the dales, across the rivers, without the least regard to any accident of the soil.

Roserl stood with her back against a massive rock, knitting away with magical swiftness, and singing with a fresh, clear, and thrilling voice. Roserl was seldom silent: if she did not talk she must sing. The woe and weal of her young life from earliest childhood upwards had been expressed in song.

As she stood there, she might have presented the picture of calm self-possessed complacency, had it not been for her eyes, which wandered down the hill in all directions, turning rather restlessly from one object to another; and that now and then a smile suddenly brightened up her face, or a shadow crossed her brow. Sometimes she would interrupt her song and stop her knitting, and attentively listen to a distant noise—the cracking of the branches in the forest below, the rustling of the wind, or the flutter of a bird; and then, with a sigh of disappointment, resume her occupation. Did Roserl except anyone?

The day had been sultry, but now light gusts of wind blew refreshingly over the Alm, and made every grass-blade tremble with delightful anticipation of coming rain.

Vivid flashes of light darted up and down the extended horizon.

Suddenly a shrill note broke upon the silence, followed by shouts—a regular, long-drawn yodler was sent up to the Alm from below.

Roserl started; her stocking fell from her hands. She drew herself up; you would have thought the girl was developing into fuller and stronger bloom. She answered the call from below. Her notes had not died away, when another shout, a wild, gay, frantically happy shout, was heard somewhat nearer than the first.

Roserl pressed her hands to her bosom; she was pale with emotion. "Franzl!" she whispered to herself; then suddenly, her face all aglow, she clapped her hands joyfully, and repeated, aloud, "Franzl! Franzl!"

A few seconds after this, the form of a young man emerged from the forest, and came bounding upwards, from rock to rock, with the speed and gracefulness of a chamois.

A tall, well-proportioned figure, with a fine manly head, adorned with a profusion of short raven locks, a healthy brown hue on the face, a splendid moustache, parted on each side, setting off a straight nose, and showing a handsome mouth, round whose corners an expression of good-humor lingered at any time, but whose lips now were parted with breathless joy. A pair of the darkest of dark eyes sent their flashing glance up the height.

He wore a green felt hat, with a bunch of artificial flowers, to which some sprigs of a forest tree, plucked in coming up, were added; a loose gray jacket over green waistcoat; the shirt tightly held together by a small black kerchief; breeches of black chamois leather, which reached to the knees, the latter remaining bare; grey worsted leggins, with a green border, and leather shoes with a double range of nails around the sole.

Juchee! Juch! cried Franzl, as with a desperate leap he cleared the last distance that separated him from his Dirnl; and alighting on the rock at Roserl's side, he waved his hat in the air.

The girl had given a little shriek at the daring jump. And now the little dissembler looked over her shoulder coquettishly up into the youth's face, and said playfully, "Ah—it is thee!—I thought it was Hansel."

"Hansel, indeed!" he answered, laughing; "didst expect Hansel?—wouldst have preferred it to be Hansel?"

"I might have waited for Hansel, as well as for thee—mighn't I?"

"*He* wouldn't have brought thee what *I* have. Look here," holding a bunch of white velvety flowers to her.

"Edelweis!" cried Roserl, delighted.

"Ay, and from the semmering too. I have brought them all the way for thee."

"Ah, thou hast been target-shooting in Styria. Of course; I remember, thou didst tell me—but I did not quite believe thee," with a touch of self-reproach. "I thought it was a joke to tease me."

"Where didst thou think I could be all the time—not coming to see thee.

"I wondered," said she, confused and blushing, busying herself with fixing the bouquet to her bodice. "And what hast thou done at the target, Franzl?" she asked, suddenly looking up again.

"Won that!" he said, taking out of his pocket a silver medal. "Juchee, it was glorious fun!" and he pirouetted on his heel with astounding dexterity, singing and waving his hat.

"Well, now!" said Roserl, "there will be no end of conceit after that."

"No, Roserl!" said he turning and seizing her hand. "Thou knowest I don't care a straw for anything, or anyone—except—thou knowest whom. If I but win thee——"

"O no!" thou thinkest thyself the only fair boy in the world, eh?" Roserl went on, jokingly, leaving her hand in his grasp, and looking mischievously into his dark eyes.

"I don't care how many boys there may be," cried Franzl: "but there is but one Dirnl, one Roserl, in the world for me." And with that he seized her round the waist, and with a loud shout of "Juchee!" lifted her from the ground, and swung her around him in a whirl.

How the two managed to keep on the little plateau, which was hardly wide enough to hold one quiet, sober person, I don't know. Franzl put Roserl on the ground again, but before releasing her, he pressed his lips to her cheek with heartiness, that you might almost have heard it resound from hill to hill.

But a slap on his cheek, not less hearty, to waken the echoes again, rewarded his audacity.

The angry expression of Roserl's face, the indignation flashing in her eyes, did not lessen her beauty; and when she said, "Oh, thou good-for-nothing, thou rascal, get thee out of my sight!" he seemed not in the least willing to obey the command. He sat down on a projecting rock on the edge of the precipice, and laughed.

Roserl stood turned from him, struggling hard to keep her dignity, until Franzl changed his tone, and rubbed his cheek dolefully.

Then her repressed merriment broke forth. "Served thee right," cried she, with a burst of laughter, patting the ill-used cheek with her small sunburnt hand, smiling saucily, and coquettishly bringing her rosy face so provokingly near to his that he could not help feeling a strong temptation to commit the offence again, whatever might come of it. However, he resisted as a man, liking her present mood too well to care to change it. He contented himself with looking at the maiden with caressing eyes; there were a thousand kisses in his glance; and the audacious girl played with the dangerous sparks as a child plays with fire.

They were chatting gay nonsense, laughing and singing in turns; he complacently sitting on the bit of rock, she standing by him, not willing to encourage him so far as to sit down beside him, keeping herself the closest possible in his grasp, and still free in her movements. How long the two foolish things went on this way they did not know, or heed.

"Roserl!" said Franzl, suddenly. He spoke softly, entreatingly, in a half whisper, for fear of rousing her temper. "Roserl!" and he said no more.

Gently, gently, he drew her head towards him, closer and closer, until it rested on his breast; then he laid his arm around her waist, scarcely touching her form, which yielded to the slightest pressure, until it was resting against his throbbing heart. But so lightly he held her, so gently he pressed her, so beseechingly his eyes rested on hers, that the girl's high spirits seemed suddenly subdued. She leant her head against his shoulder, and her sweet eyes met full and frankly an ocean of honest love beaming from his.

The wind blew fiercer over the mountains; the noiseless flashes lighting up the heavy mass of dark clouds, began to find utterance in low growling of distant thunder.

"Roserl!" whispered Franzl, passionately. "My own Roserl!"

There was a strange force in that word "Roserl!" It was worth all the eloquence of a lover's protestation. And Roserl's answer to his appeal was yet simpler: she did not say a word.

But did not her eyes speak volumes?

Franzl had often looked deeply into those beautiful orbs; he was familiar with every change in their expression, but never had he seen heaven itself in them, opening upon him as he saw it now. She had no need to speak.

"Roserl—my sweet, my own Roserl, thou art surely mine!" Their embrace became closer, until heart beat to heart, lip rested on lip, with fond, passionate clinging.

The glaring flashes of light followed by peals of thunder, the rising hurricane, did not disturb the lovers' rapture. More fondly clinging to each other as the storm approached, they lost all perception of things around them.

A vivid flash, changing for one moment the night into something like brightest moontime, a tremendous thunderclap, and a sudden shower of rain, tore them asunder at last.

Roserl had started first. She felt as one awaking from a dream, and looked ashamed of herself the next moment, then half angry, then something like herself again. Franzl seemed not so willing to be roused from a dream of heavenly bliss; he did not stir at first; then slowly rising, he took Roserl by the hand, and said, solemnly:

"Roserl, thou promised to be my wife?"

"Yes," said she, without the least hesitation.

The shower had not lasted; the wind seemed too high to admit of rain; the heavy drops even were turned away; the black masses of clouds moved rapidly; lightning and thunder continued.

"I forgot my cows!" cried Roserl, in self-reproach, and off she bounded, Franzl following.

They shut the wide gate upon the melancholy bellowing cows, who seemed to complain of neglect, and closed every opening on the weather side.

"The poor beasts must feel it warm in here, I am sure," said Roserl. "I couldn't stop in my room on a stormy night. Ha! what a flash! what thunder! Come up here, Franzl. How it shakes the earth! Here we can best see it. What a spectacle! Hast thou ever seen a more splendid sight? For nothing in the world would I miss my summer's stay on the Alm. Ah see—see!"

"*For nothing in the world?*" cried Franzl, with apprehension. "Oh, Roserl, married women do not generally go up as sennerin."

"No—o," said Roserl, and her countenance fell. "I suppose it would not do. But I am not to be a married woman all at once. Surely there is no hurry, Franzl. I don't want to be married for another year or two—I am so young—we are both young; surely we can wait." This was said so innocently and prettily, with so charming a grace, that Franzl, in spite of his feeling of disappointment, could but reply:

"No; there is no hurry, of course; we need not be married at once; but we can fix some future time——"

"Oh, Franzl!" cried Roserl, coaxingly, "thou knowest I am thine, nothing can part us; but there is no hurry to fix the time; it will come round by itself sure enough. In the meantime——"

"Oh, I am happy enough in the prospect of having thee for my wife in some future unknown time," interrupted Franzl. "I will be patient, if I am sure of my prize in the end."

"Of course. I would not marry any one else in the end."

"But if I am to wait for thee, I must in the meantime see thee often—always."

"Of course."

"Every day?"

"Twice a day, if thou choosest."

"But, Roserl."

"What, Franzl?"

"When I come thou must receive me kindly."

Roserl laughed.

"Not as thou didst receive me to-day."

Roserl laughed again. Poor boy! Then added, tenderly: No more cuffs for thee, Franzl."

"Dost thou mean what thou sayest, Roserl?"

"Art thou not my own, my proud exacting Franzl?" and taking his hand in both hers: "I will always receive thee kindly!"

"With sweet kisses?"

"Oh, the impudence!" cried Roserl, letting his hand fall from her grasp. "No."

"Why not, Roserl? Art thou not mine?"

It might occur to Roserl that she had gone too far in denying every fond caress in the future, and her face happening to be close to his shoulder, he having his arm round her—for fear she might be blown away by the tempest—she managed to whisper in his delighted ear: "I will tell thee one thing. Listen! Thou shalt come to see me every day, I say; I will not take any excuse; and I—I will give thee a kiss at thy coming—and again kiss thee once—*once*, I say—at parting. Dost thou agree to this?" And she released herself from his arm.

"And if I am not agreeable?"

"Then I won't have thee up here at all!"

"If it is so, I must agree, of course. But thou wilt think better of it, my Roserl; thou wilt not be so miserly with thine alms!"

"Yes or no?"

"Thou grantest very little!"

"Yes or no?" growing impatient.

"Before we settle the contract, Roserl, I must have a specimen of those promised two kises; give me a sample, Roserl."

Roserl laughed and blushed and winced, and after a little struggle she sprang forward, and throwing her arms round his neck, she held her full cherry lips temptingly up to him. Then she succeeded in extricating herself from his encircling arms, and took her stand a few steps distant from him. She looked blooming, agitated, but happy. "Dost thou agree, Franzl?"

"I think I must," said Franzl, looking at her with hungry eyes. "I fear I must. But, Roserl, thou dost not know———"

"Wilt thou agree?"

"So very little, Roserl!"

"No more!" cried Roserl, her patience fairly worn out. "I

know somebody who would be happy if I allowed him to kiss my little finger once in my life!"

"Hansel, of course?"

"*He* wouldn't grumble."

"He cannot love thee as I do."

"Dost imagine so?"

"But it is of no consequence whether he does or does not. He must not come to see thee here, Roserl."

"Must not!" said Roserl, in an aggravated tone.

"What meanest thou, Roserl?"

"That Hansel won't give up coming to see me."

"Unless thou tellest him not to come."

"But I won't tell him that."

"Roserl!"

"I won't."

"My own Roserl."

"Well?"

"If thou art to be my wife, thou must as a matter of course give up Hansel."

"But not until I *am* thy wife. In the meantime I expect to be free to amuse myself as I please."

"But, Roserl, if thou carest for me, thou canst not care for Hansel."

"I care for him just enough to want to see him. He amuses me, he brings life up to the Alm; we couldn't do without Hansel. Every girl envies my seeing so much of him; it is great fun to witness their jealousy!"

Franzl felt perplexed. "But, Roserl," he stammered, "when people once know of our engagement——"

"They won't know. I won't tell them, and I won't have thee tell them. I *will* have my sommerfrische out without being talked about."

"But if Hansel——"

"Isn't it enough that *thou* knowest I love thee best? What does it matter to us what other people think?"

"Hansel and I will meet here; and he seeing that I come every day, will come every day too," said Franzl, quite bewildered.

"You must both take your chance," quoth the girl, haughtily.

"I cannot understand," said Franzl, pensively, how thou canst care to see Hansel here——"

"How?" she interrupted, with animation. "Isn't he the merriest boy hereabouts? A little too wild, perhaps, but the more fun. If I didn't but love another best"—half crying—"an ungrateful wretch, I might well be proud to be chosen by Hansel."

"And in thy estimation there is no merrier youth in the world? Sapperment!"

"Isn't he the best shot hereabouts? The bugbear of the Forster? Thee will never catch Hansel, poor old man, he is too clever!"

"Roserl!" cried Franzl, speaking with emotion, "why dost thou say this? Thou knowest well there are others who can point a shot as sure as Hansel, if not better; and could do, and *have* done the same exploits as he. Dost thou forget but for the accident two years ago, when I promised my dying mother never to wildern again, I could outdo Hansel any day?"

"Of course," said the girl, softened, "that was a misfortune, to shoot one's mother's brother nearly dead. He had a narrow escape."

"And after all, Roserl, It is not respectable to be a wildschutz. It is against the law. Thou oughtest to be glad that thy lover has tried a hand at it to show what he can do, and has given up the dangerous pastime."

"Somehow I can't be glad at that."

"Roserl" hissed Franzl, with a strange huskiness in his voice, "thou couldst almost tempt a man to break his most sacred vow."

"Nobody tempted thee. Did I?"

"It seems the only way to win thy regard. Now if I were free to outdo Hansel in wild hunting, thou wouldst not be ashamed to acknowledge thy preference of me!"

There came a pretty little shrug of her shoulder as her doubtful answer.

"If there were a grain of love for me in thy heart——"

"If thou choosest to doubt it——" She could not continue; she all but wrestled with her wrath.

They had both managed to work each other into an ungovernable passion.

"Look here," cried Franzl, with sudden determination. His lips were white with suppressed agitation. "What's the use of wrangling?" Thou must choose between us; we both cannot court thee. If thou hast chosen me—as I had a right to believe a little while ago—it would not be fair towards him to let him go on losing his time after all."

"Fortunately for Hansel, he doesn't regret his time lost upon *me*," said Roserl. "He would give his life to see me for no more than twenty yards off. Very different from some others."

Franzl took no notice of the last hint, and continued: "Because he hopes still to make thee love him some day. He cannot know that all shame is lost. How can he suspect such double play? Roserl, it is wrong."

But now the girl's hardly restrained wrath fairly broke out. "What!" screamed she, "teach me wrong or right—darest thou? Now, before we are married! What wouldst thou do after our marriage?" "Remember," she panted, 'we are not man and wife yet; perhaps never shall be. No, no! I won't be tied to——" Here her voice broke down: her whole frame shook; her eyes flashed in rage; her lips were white and trembling. Was this the same girl who but a few minutes before had nestled lovingly on Franzl's heart? the same sweet eyes so divinely shining into his? the same fond lips upraised to meet his kiss? Ah, me! all that had been a little, very little, while ago. So short had been the time that had wrought the change, that Franzl was bewildered, beyond himself with surprise. Grief, rage, awe, mingled in his breast, found expression in his look.

The weather seemed to rage in common accord with them. Flash after flash, peal after peal, showers of rain in the roaring hurricane. But the two were now beyond noting that they had become wet through.

"What dost thou mean by 'never be'?" cried Franzl.

"That I won't be bound to such a tyrant!"

"Tyrant!" shouted he, above the wind—and stopped to rally his strength. "Mark my words," with great stress, "*thou must give up Hansel* or *me! I* won't be a traitor towards a friend. I will not suffer *him* to be wronged. Who is it to be—*him* or *me?*"

"Get thee hence, and that instantly!" cried the girl, at the top of her voice; "and mark my words now: *I throw thee off as I do these flowers*—there they go, and all my love for thee departs with them from me—goes with them down to the bottom of the preci pice!" Thus saying she vehemently plucked Franzl's boquet from her heaving breast, raised her arm high, and flung it far away from her. Down, down it went into the great dark gulf.

A deadly palor spread over Franzl's face. He sprang to the brink of the precipice. His tone was icy, his teeth chattered, as he replied: "Very well; it won't be more difficult to bring the flowers out of the hole than it was to bring them from the semmering When I have brought them back again, I will give them to Feferl!"

His words sounded like a death-knell, as he disappeared the way the flowers had gone down before him.

(TO BE CONTINUED.)

